# Genetic Polymorphism Effect on Warfarin–Rifampin Interaction: A Case Report and Review of Literature

**DOI:** 10.2147/PGPM.S288918

**Published:** 2021-01-26

**Authors:** Muhammad Salem, Islam Eljilany, Ahmed El-Bardissy, Hazem Elewa

**Affiliations:** 1 Department of Pharmacy, Hamad General Hospital, Doha, Qatar; 2 College of Pharmacy, QU Health, Qatar University, Doha, Qatar; 3 Biomedical and Pharmaceutical Research Unit, QU Health, Qatar University, Doha, Qatar

**Keywords:** warfarin, rifampin, interaction, *CYP2C9*, *VKORC1*, genotype

## Abstract

Warfarin–rifampin interaction has been reported since the 1970s. Due to rifampin’s strong induction of *CYP2C9*, most cases could not attain the target international normalized ratio (INR) despite warfarin dose escalation. Genetic polymorphisms determine up to 50% of warfarin dose variability. A 38-year-old woman was started on warfarin and rifampin for cerebral venous sinus thrombosis and pulmonary tuberculosis. Over six weeks, the daily warfarin dose was increased from 3 to 10 mg to attain three consecutive in-clinic therapeutic INRs. She completed three complications-free months of warfarin treatment with time in therapeutic range (TTR) of 46%. We performed retrospective genetic testing to determine the patient’s *CYP2C9, CYP4F2*, and *VKORC1* genotypes and whether they had affected the interaction outcome. The analysis revealed that the subject carries CYP2C9*3*3 and *VKORC1-1639 (GA)* mutations, classifying her as a slow metabolizer and, hence, highly warfarin-sensitive. This was reflected on how the case responded to a relatively lower dose than previously reported cases that did not achieve the target on warfarin daily doses up to 35 mg. This is the first report addressing the genotype effect on this interaction. Patients with genetic variants requiring low warfarin doses are more likely to respond at a feasible dose while on rifampin. Future studies to evaluate warfarin–rifampin-gene interaction are warranted.

## Introduction

Warfarin, a vitamin K antagonist, remains the preferred oral anticoagulation for atrial fibrillation with prosthetic cardiac valves or rheumatic heart disease with more than mild mitral stenosis, and venous thromboembolism (VTE) at unusual sites.^1^,^2^ It is a mixture of two racemic isomers, *R* and *S*-warfarin. Both impair the vitamin k-dependent proteins production via inhibition of vitamin K epoxide reductase complex subunit 1 (VKORC1).^3^ Cytochrome P450 2C9 (CYP2C9) extensively metabolizes *S*-warfarin, the stereoisomer of predominant potency, to the inactive 7-hydroxywarfarin.^3^ *VKORC1* and *CYP2C9* genetic polymorphisms, with other genetic variants, determine up to 50% of warfarin dose variance.^3^,^4^ The most studied and common variant alleles of *CYP2C9* are CYP2C9*2 *(rs1799853)* and *3 (rs1057910), which result from missense mutations associated with diminished catalytic activity, poor warfarin metabolism, and decreased dose requirements.^5^ *VKORC1–1639G>A (rs9923231)* genotype variants (*GA* and *AA*) contribute majorly to sensitizing warfarin.^5^ On the other hand, CYP4F2*3, a nonsynonymous variant of the gene coding for the primary liver vitamin K oxidase, CYP4F2, has been associated in some studies with a modest increase of warfarin dose requirements (8–11%).^3^ These mutations were incorporated, with other clinical factors, into dosing algorithms which were shown to provide better warfarin dose prediction.^3^ Gage and International Warfarin Pharmacogenetics Consortium (IWPC), are among the most widely studied algorithms and are contained in the website (www.warfarindosing.org), which calculates the initial dose with the ability to adjust for CYP2C9*5, *6, CYP4F2, and *GGCX* genotypes.^3^ Genetic-based dosing of warfarin upon initiation was shown in some studies to improve target international normalized ratio (INR) attainment and time in therapeutic range (TTR) during the initial month.^6^ This was mainly mediated by *CYP2C9* and *VKORC1* polymorphisms which significantly impact the maintenance dose requirement.^3^,^4^,^7^ The Food and Drug Administration (FDA) has also approved warfarin label modifications with dosing guidance based on the *CYP2C9* and *VKORC1* genotypes.^8^ Apart from genetics, numerous warfarin drug interactions have been reported requiring dose adjustments and frequent INR monitoring to avoid bleeding or anticoagulation failure.^9^

Antituberculous management includes rifampin, isoniazid, ethambutol, and pyrazinamide. While ethambutol and pyrazinamide are neither cytochrome P450s inhibitors nor reported to affect warfarin, isoniazid is a week inhibitor of CYP3A4, which is not FDA classified as a clinical index inhibitor.^10^ While it has been reported to increase warfarin’s hypoprothrombinemic effect in two cases,^11^,^12^ the interaction magnitude is considered minimal, with no action recommended.^13^

Rifampin, a life-saving antimicrobial for tuberculosis, endocarditis, and meningitis,^14^ is a clinically significant inducer of CYP2B6, CYP2C19, CYP2C8, CYP2C9, and CYP3A4, as well as P glycoprotein.^10^ It induces CYP2C9 transcription by binding to its main De-novo synthesis regulatory nuclear receptor, pregnane X receptor (*PXR*).^5^,^15^ The binding increases the *CYP2C9* mRNA expression by up to six-folds.^15^ That leads to a higher amount of the enzyme, and extensive metabolism of the substrate/victim drug.^15^ While the onset of induction can be few days with rifampin,^16^ the time required to reach maximal enzyme abundance and new steady state is more than two weeks based on the CYP turnover and degradation half-life.^16–19^ FDA classifies rifampin as a moderate inducer of CYP2C9, defined as a decrease in the substrates area under the concentration-time curve (AUC) by 50% to less than 80%.^10^ That was based on two healthy-volunteers studies with probe substrates, *S*-warfarin and tolbutamide.^20^,^21^ However, the first study’s duration was only four days,^20^ and 12–15 days in the latter,^21^ which, yet, showed high variability of CYP2C9 activity (1–7.4-fold).^22^ These short durations may imply that rifampin is rather a strong CYP2C9 inducer as AUC ratio were measured before reaching maximal induction.^16–19^

Rifampin almost eliminates warfarin’s therapeutic effect, which required extensive dose escalation in all cases and is typically associated with the inability to maintain therapeutic range. The interaction has been described since the 1970s.^20^,^23^,^24^ Till the late 1980s, multiple reports showed a significant increase in warfarin dose requirements with rifampin.^25–27^ After the INR test was universally adopted,^28^ several reports demonstrated that most patients who required the anti-infective along with anticoagulation were unable to maintain target INR.^14^,^29–36^

This report aims to describe a case who received warfarin and rifampin concomitantly and the interaction outcome, and to perform genetic testing to determine the patient’s *CYP2C9, VKORC1, CALU*, and *CYP4F2* genotypes and whether they could explain the response to warfarin dose escalation.

## Case Description

A 38-year-old Ethiopian woman with a weight of 60 kg, a height of 150 cm, and a history of diabetes and immune thrombocytopenic purpura (ITP) on metformin and chronic eltrombopag presented to the emergency of Hamad General Hospital in Qatar on January 13, 2020, with dizziness, severe diffuse headache, photophobia, and multiple vomiting for three days. Intracranial computed tomography (CT) venogram showed cerebral venous sinus thrombosis (CVST). Eltrombopag was stopped. Since the Glasgow Coma Score (GCS) dropped to 11, the patient was admitted to the medical ICU with sedation, analgesia, and close neurologic observation. Because of low platelet count (PC), 32X10^9^/L, she was started on 0.5 gm/kg intravenous immunoglobulin (IVIG) plus steroids for three days to raise the PC above 50X10^9^/L in order to initiate anticoagulation. Two days later, PC reached 75X10^9^/L, and heparin continuous IV infusion was initiated with platelets transfusion as the patient was neurologically deteriorating, and repeated CT showed extension of thrombosis with intracerebral hemorrhage (ICH) and subarachnoid hemorrhage (SAH). On January 19, although PC was maintained above 200X10^9^/L, heparin was suspended due to a sharp drop of hemoglobin to 5 gm/dL with no identified source of bleeding. The patient was transfused immediately. The next day hemoglobin increased to 8 then maintained at 9–10 gm/dL. Heparin was resumed on January 23. Hematology planned to start rituximab for ITP, yet the Quantiferone test for tuberculosis (TB) was positive. CT chest on January 26 revealed consolidation patches in the right upper and middle lung lobes suggesting active TB. Since bronchoscopy was not feasible due to the high bleeding risk, the pulmonary, MICU, and infectious diseases teams decided to start empiric anti-TB medications based on radiology and follow response by imaging. The rituximab plan was aborted.

**Figure 1:**
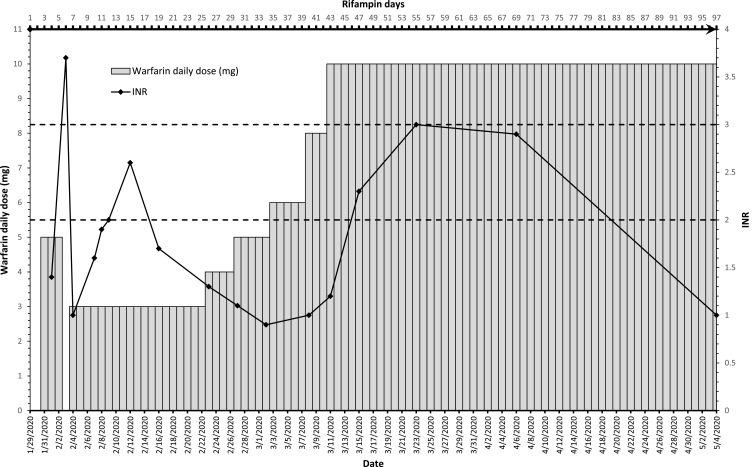
This graph represents the daily warfarin dose, rifampin days, and INR overtime. The bottom x-axis represents dates. The top x-axis represents rifampin days. The left y-axis represents the daily warfarin dose in milligrams and is shown by the vertical bars. The right Y-axis represents the INR and is shown by the black diamond points. The therapeutic range is indicated between the two dotted lines (2.0–3.0).

On January 29, the patient was started on daily rifampin 600 mg, isoniazid 300 mg, ethambutol 1100 mg, and pyrazinamide 1600 mg. She was transferred to the medicine ward after sedation withdrawal. On January 31, anticoagulation was shifted to daily oral warfarin 5 mg with twice-daily enoxaparin 60 mg as bridging. Three days later, warfarin was held for one day then resumed with dose reduction to 3 mg due to a sharp INR increase to 3.7. The next day, INR dropped to 1.0, then increased gradually after three days to 1.7, 1.9, and reached 2.0 on February 9. Enoxaparin was stopped, and the patient was discharged on warfarin 3 mg, anti-TB medications, pyridoxine 40 mg, metformin 500 mg twice daily, and metoprolol 50 mg twice daily. Following in anticoagulation clinic, on February 12, after 14 days of rifampin, INR was 2.6; therefore, the same warfarin dose continued. Although decreased to 1.7 on February 16, the warfarin dose remained. A week later, INR dropped to 1.3, so warfarin was escalated to 4 mg/day, and enoxaparin resumed. Over the next three weeks, the dose was gradually escalated up to 10 mg, after four days of which, INR reached 2.3 on March 15. Enoxaparin was stopped, and the patient was maintained on daily warfarin 10 mg. The anti-TB medications were switched to (Rifampin 600 mg/Isoniazid 300 mg) on March 24. INR was maintained in the next two clinic visits at 3 and 2.9 on March 23 and April 6, respectively. On May 4, the INR was 1.0 in the last anticoagulation clinic visit. That could not be explained by non-compliance as per the patient interview. The planned three-month duration of anticoagulation ended, so warfarin was stopped. Rifampin/isoniazid continued till July 14, 2020. The patient was interviewed on each visit and excluded any adverse effects. She had normal follow up laboratory values throughout the treatment. Details of warfarin daily dose and INR are shown in [Figure 1].

## Methods

The patient was approached by one of the study investigators and explained the reasons and expectations of the research. The patient confirmed her understanding, and agreement to provide saliva sample for genetic testing as well as to have the case published by signing an Institutional Review Board (IRB) approved informed consent form. She was asked to provide a saliva sample using Oragene•DNA (OG-500) self-collection kit (DNA genotek, USA). Hereafter, the kit was kept in a water bath at 50 C° overnight for DNA extraction. The prepIT^®^•L2P standard protocol for the purification of DNA was used for DNA extraction.^37^ The purified DNA’s quality and quantity were evaluated by Nanodrop 2000c Spectrophotometer (Thermo Fisher Scientific). Finally, the sample was genotyped for detecting the following genetic variants: CYP2C9*2 (rs1799853), CYP2C9*3 (rs1057910), CYP4F2*3 (rs2108622),VKORC1 (rs9923231), VKORC1 Asp36Tyr (rs61742245), and *CALU (rs339097)*. This was performed using the QuantStudio™ 5 Real-Time Polymerase Chain Reaction (PCR) system for Human Identification, 96-well, 0.2 mL, desktop manufactured by Applied Biosystems^TM^.

## Results

The genotyping revealed that the patient is a carrier of CYP2C9*3*3 homozygous, *VKORC1-1639 (GA)* heterozygous, and *CYP4F2 (CC)* wild-type homozygous. Based on this genetic profile, the subject is considered a slow metabolizer which indicates high warfarin sensitivity. On the other hand, both *VKORC1 Asp36Tyr (CT)* and *CALU (AG)* genotyping were heterozygous indicating partial warfarin resistance.

As it is shown in Figure 1, the sudden rise in INR with a moderate standard warfarin dose of 5 mg is very well explained by the *CYP2C9* loss of function genetic variant carried by this patient (CYP2C9*3/*3). A few weeks later and with the interacting effect of rifampin reaching its peak, the daily warfarin dose requirement for the patient increased reaching 10 mg.

## Discussion

In this case report, we observed warfarin–rifampin drug interaction in a patient with CVST and pulmonary TB. The interaction management required warfarin dose-escalation, frequent INR monitoring, and low molecular weight heparin (LMWH) bridging over six weeks. On a daily warfarin dose of 10 mg, our patient attained three consecutive therapeutic INR levels in three clinic visits. That is considered a “stable warfarin dose,” as defined in most studies,^38^ despite the one subtherapeutic INR in the last treatment day. The TTR, calculated by the Rosendaal method,^39^ was 46.2%. The three-month warfarin anticoagulation treatment, combined with rifampin, was completed with difficulty, yet no complications.

Since the World Health Organization (WHO) adopted INR in the 1980s,^28^ more than nineteen cases of warfarin–rifampin interaction have been reported.^14^,^29–36^ Despite extensive warfarin dose escalation over a prolonged time, the majority could not attain target INR while on the combination.^14^,^29–32^,^34–36^ Cases are summarized in [Table 1]. Excessive warfarin exposure and hemorrhage after rifampin discontinuation have been reported, implying the importance of close monitoring and careful dose de-escalation after rifampin stoppage.^33^,^40^ Due to the lack of laboratory monitorable parameters like INR, labels of direct oral anticoagulants (DOACs), that are substrates of CYP3A4 and/or P glycoprotein, recommend avoiding concomitant use with rifampin to avoid unmanageable therapeutic failures.^41^

**Table 1:** Summary of Studies of Warfarin–Rifampicin Interaction with INR Monitoring

Study	Patient’s Data				Sequence and Duration		During Concomitant Use							After Rifampin Stopped	
	Case No.	Age	Sex	Warfarin Indication	Rifampin Start in Relation to Warfarin Start (Days)	Concomitant Duration (Days)	Average Initial Warfarin Dose (mg/day)^a,b^	Average Last Warfarin Dose (mg/day)^a,b^	Warfarin Dose Increase	Target INR	Target Attained	Time to Stable Warfarin Dose^c^ (Days)	TTR	Stable Warfarin Dose^c^ (mg/day)	Time to Stable Warfarin Dose^c^ (Days)
Casner, 1996^29^	1	36	M	PE	−2	20	7.8^b^	20^b^	169%	2.0–3.0	No	NA	NA	10	10
Lee & Trasher, 2001^30^	1	58	M	LVT	−120	113	7.5^b^	25^b^	233%	2.0–3.0	No	NA	NA	7.5	35
Kim et al, 2006^31^	1	79	M	DVT	−11	30	5^a^	30^b^	500%	2.0–3.0	No	NA	NA	6	60
Krajewski, 2010^32^	1	71	M	AF	+14	45	5.7^a^	25^b^	340%	2.0–3.0	No	NA	NA	5	120
Martins et al, 2013^33^	1	59	F	AF	+300	203	6.4^a^	11.4^a^	78%	2.0–3.0	Yes	104	50%	5.4	60
Maina et al, 2013^36^	1	17	F	DVT	−7	UNK	10^b^	27.7^a^	177.3%	2.0–3.0	Yes	63	52%	UNK	UNK
	2	24	F	RHD/LAT	−42	UNK	5^b^	5.8^a^	16%	2.0–3.0	Yes^d^	66	67%	UNK	UNK
	3	36	M	DVT	−44	UNK	12^b^	11.4^b^	−4.8%	2.0–3.0	No	NA	24%	UNK	UNK
	4	64	F	DVT	−45	UNK	10^b^	11.5^b^	15.3%	2.0–3.0	No	NA	47%	UNK	UNK
	5	22	F	DVT	−88	UNK	10^b^	4.8^a^	−37%	2.0–3.0	Yes^e^	12	54%	UNK	UNK
	6	9	M	DVT	0	UNK	5^b^	5.3^b^	5.8%	2.0–3.0	No	NA	53%	UNK	UNK
	7	49	M	DVT	−3	UNK	5^b^	9.5^a^	89.3%	2.0–3.0	Yes	49	42%	UNK	UNK
	8	30	F	PE	−35	UNK	5^b^	27^a^	440.9%	2.0–3.0	Yes	67	30%	UNK	UNK
	9	29	F	DVT	−31	UNK	5^b^	11.8^a^	135.8%	2.0–3.0	Yes	7	40%	UNK	UNK
	10	41	M	Stroke & DVT	−46	UNK	6^b^	6.5^a^	8.3%	2.0–3.0	Yes	63	66%	UNK	UNK
Dawson et al, 2016^34^	1	60	M	MV replaced	+UNK	42	8^a^	35^b^	340%	2.5–3.5	No	NA	NA	8	28
Fahmi et al, 2016^14^	1	34	F	MV replaced	+19 years	42	7.5^a^	30^b^	300%	2.5–3.5	No	NA	NA	11.4	35
Shibata et al, 2017^35^	1	70	F	Stroke	+UNK	365	4^a^	15^a^	275%	2.0–3.0	Yes	UNK	UNK	4	30
	2	80	F	AF	+UNK	330	2.5^a^	10^b^	300%	2.0–3.0	No	NA	NA	3	60

**Notes:** Maina et al did not report rifampin cessation times or confirmed adherence to medications other than warfarin,^36^ which may have affected response. ^a^Therapeutic INR attained on the dose. ^b^Therapeutic INR not attained on the dose. ^c^Defined as three consecutive therapeutic INR levels on the same warfarin dose (In Maina et al case series, defined as two consecutive therapeutic INRs). ^d^Occasional warfarin overdoses. ^e^Missed warfarin doses.

**Abbreviations:** AF, atrial fibrillation; DVT, deep venous thrombosis; INR, international normalized ratio; LAT, left arterial thrombosis; LVT, left ventricular thrombus; MV, mitral valve; NA, not applicable; PE, pulmonary embolism; RHD, rheumatic heart disease; TTR, time in therapeutic range; UNK, unknown.

Our case had one supratherapeutic INR after three warfarin doses of 5 mg and five days of rifampin commencement explained by her *CYP2C9* poor metabolizer phenotype. She had two consecutive therapeutic INR readings after 12 and 14 days of rifampin on daily warfarin 3 mg. However, INR dropped below therapeutic after 17 days of rifampin, likely due to the latter’s liver enzyme induction. Comparable patterns have been observed in other cases when the commencement sequence was a few days apart or when rifampin was added to chronic warfarin. In 1996, Casner^29^ reported a patient who had one therapeutic INR after 13 rifampin days, which declined to subtherapeutic until ten days after the rifampin stoppage. The case reported by Kim et al^31^ had one in-target INR after warfarin 20 mg, which dropped then increased back to target for a week after escalation to 25 mg on rifampin days 25 and 33. Then, the INR declined steadily even after warfarin dose was raised to 30 mg.^31^ An INR of 4.4 after three days of rifampin in a mechanical valve replacement case, reported by Fahmi et al^14^, necessitated the holding of warfarin for five days. Then INR dropped to below the desired 7–14 days from the combination. In the case reported by Dawson et al^34^, it took 17 days for the interaction to become apparent. These reports, aligned with our report, indicate that the time-course to rifampin CYP2C9 induction is more than 14 days. Studies of this rifampin duration or less are inadequate to evaluate the full impact of such interaction. It is vital to monitor INR frequently during the first two weeks of rifampin and shortly after to avoid exposing the patient to subtherapeutic levels with no bridging.

Our patient’s *CYP2C9* genotyping revealed that she is a slow metabolizer with a homozygous CYP2C9*3*3, which indicates high warfarin sensitivity and the lowest dose requirements. Additionally, *VKORC1 –1639G>A* genotyping showed upstream variant *(AG)* requiring lower warfarin doses.^3^ The patient reached three consecutive therapeutic INRs on a daily warfarin dose of 10 mg, which is generally considered a high dose. However, compared with most cases that received rifampin with warfarin doses up to 35 mg and no target attainment, 10 mg represents a relatively reasonable dose. The estimated therapeutic warfarin dose for our case, calculated using clinical factors via www.warfarindosing.org, was 5.8 mg/day. However, when the genotyping results were added to the calculation, the estimated therapeutic dose was 2 mg/day. The FDA warfarin label expected maintenance dosing for CYP2C9*3/*3 combined with *VKORC1 (AG)* is ranged from 0.5 to 2 mg/day.^8^ That implies that 10 mg is at least a 400% increase from the therapeutic dose without rifampin. Interestingly, most reported cases who reached therapeutic INR during the concomitant warfarin–rifampin use received relatively low warfarin doses around 10 to 15 mg/day.^33^,^35^,^36^ Since the reported cases were not tested for genotype variations; genetic polymorphism might represent an unrevealed explanation of the variable warfarin responses among patients during rifampin use.

Up to our knowledge, this is the first warfarin–rifampin interaction case report to address genetic polymorphism as a contributing factor in the interaction outcome since it was first described in the 1970s. Nevertheless, there are studies of rifampin induction effect on *CYP2C9* variants. Vormfelde et al^42^ used tolbutamide as a probe substrate to evaluate rifampin effect on CYP2C9 activity in 128 healthy volunteers with different genotypes. While the pre-rifampin enzyme activity difference between *1/*1 and *3/*3 was six-folds, the induction effect was around two-fold regardless of the genotype.^42^ It is important to emphasize that the study duration was only four days,^42^ which may not represent the subsequent enzyme induction phases. George et al^43^, using phenytoin as a probe drug, studied the total effect of one month of rifampin therapy on *CYP2C9* various genotypes in forty-eight new TB patients. Joined data from twelve mutant patients showed that rifampin’s induction potential was statistically significant regardless of the genotype.^43^ While these studies suggest that rifampin increases the CYP2C9 concentration with the same ratio,^42^,^43^ the catalytic activity would be genotype-dependent.^5^

Our case may represent an example of phenoconversion, a phenomenon of genotype-phenotype mismatch, in which an individual’s metabolizing enzyme is functionally converted from a poor metabolizer to an intermediate or extensive metabolizer or vice versa due to the use of an enzyme inducer or inhibitor, respectively.^44–46^ Rifampin shifted the patient from her genotype-based poor metabolizer status to a functional rapid metabolizer status that required warfarin daily dose escalation.^46^ However, because the patient’s gene-based estimated warfarin dose was 2 mg, escalation to 10 mg, a feasible dose compared with other interaction reports, was sufficient to attain therapeutic INR.

While the patient confirmed complete adherence, the INR dropped to 1.0 on the last day of warfarin therapy and day 97 of rifampin. One explanation may be a late CYP2C9 induction phase by rifampin. CYP2C9 half-life has been reported to be much longer than other CYPs as CYP3A4. Shibata et al^35^ monitored CYP2C9 and CYP3A activities in two cases who were receiving rifampin and warfarin concomitantly after rifampin discontinuation. The CYP2C9 estimated half-lives were 25.7 and 16.8 days, compared with CYP3A half-lives of 2.4 and 11.5 days, in the first and second case, respectively.^35^ Indicating that the CYP2C9 turnover can take up to months. Moreover, having a CYP2C9*3*3 genotype might have prolonged the time-course to maximal induction. Since warfarin was stopped at that point, verifying these explanations is not possible.

## Conclusion

This case report demonstrated the highly significant effect of rifampin metabolic induction and genetic polymorphism on warfarin dose requirements. Our findings reveal a genetic explanation of the variable patients’ responses to different warfarin doses while on rifampin. While wild-type patients are not likely to respond to extreme warfarin doses due to the drug interaction with rifampin, patients with loss-of-function genetic variants of *CYP2C9* and *VKORC1* are more likely to respond at a feasible dose level. Future studies are warranted to evaluate the genotype variants’ effect on the interaction, which might benefit in selecting likely warfarin responders when rifampin therapy is needed.
